# Assessment of Vascular Network Connectivity of Hepatocellular Carcinoma Using Graph-Based Approach

**DOI:** 10.3389/fonc.2021.668874

**Published:** 2021-07-06

**Authors:** Qiaoyu Liu, Boyu Zhang, Luna Wang, Rencheng Zheng, Jinwei Qiang, He Wang, Fuhua Yan, Ruokun Li

**Affiliations:** ^1^ Department of Radiology, Tenth People’s Hospital of Tongji University, Shanghai, China; ^2^ Department of Radiology, Ruijin Hospital, Shanghai JiaoTong University School of Medicine, Shanghai, China; ^3^ Institute of Science and Technology for Brain-Inspired Intelligence, Fudan University, Shanghai, China; ^4^ Department of Radiology, Shanghai Chest Hospital, Shanghai JiaoTong University, Shanghai, China; ^5^ Department of Radiology, Jinshan hospital, Fudan University, Shanghai, China

**Keywords:** hepatocellular carcinoma, vascular network, topologic connectivity, Micro-CT, graph-based approach

## Abstract

**Background:**

The angiogenesis of liver cancer is a key condition for its growth, invasion, and metastasis. This study aims to investigate vascular network connectivity of hepatocellular carcinoma (HCC) using graph-based approach.

**Methods:**

Orthotopic HCC xenograft models (n=10) and the healthy controls (n=10) were established. After 21 days of modeling, hepatic vascular casting and Micro-CT scanning were performed for angiography, followed by blood vessels automatic segmentation and vascular network modeling. The topologic parameters of vascular network, including clustering coefficient (CC), network structure entropy (NSE), and average path length (APL) were quantified. Topologic parameters of the tumor region, as well as the background liver were compared between HCC group and normal control group.

**Results:**

Compared with normal control group, the tumor region of HCC group showed significantly decreased CC [(0.046 ± 0.005) *vs.* (0.052 ± 0.006), *P*=0.026], and NSE [(0.9894 ± 0.0015) *vs.* (0.9927 ± 0.0010), *P*<0.001], and increased APL [(0.433 ± 0.138) *vs.* (0.188 ± 0.049), *P*<0.001]. Compared with normal control group, the background liver of HCC group showed significantly decreased CC [(0.047 ± 0.004) *vs.* (0.052 ± 0.006), *P*=0.041] and increased NSE [0.9938 (0.9936~0.9940) *vs.* (0.9927 ± 0.0010), *P*=0.035]. No significant difference was identified for APL between the two groups.

**Conclusion:**

Graph-based approach allows quantification of vascular connectivity of HCC. Disrupted vascular topological connectivity exists in the tumor region, as well as the background liver of HCC.

## Introduction

Hepatocellular carcinoma (HCC) is a hypervascular tumor characterized by neoangiogenesis, which contributes to the high rate of metastasis and dismal prognosis (1). On microscopic observation, HCC displays marked geometric and structural vascular abnormalities, arteriogenesis, and capillarization (2). Accurate quantification of microvessel characteristics may help clarify biological characteristics and effectiveness of anti-angiogenic therapy (3).

So far, immunochemistrical microvessel density (MVD) is still the most accepted and applied index for the measurement of HCC vascularization, in which high tumor neovascularization often represents high invasiveness. However, MVD measurement is limited by its invasive nature, determination of hotspot, counting methods, and the inability to fully capture the vascular trajectory and tissue complexity in the entire tumor (4, 5). Vascular pattern heterogeneity also plays a role in tumor progression. For example, vessels that encapsulate tumor clusters (VETC) have continuous branches and an apparent lumen, which was linked to HCC metastatic dissemination, early recurrence, shorter disease-free survival, and overall survival (6). Additionally, current indicators for identification of MVD are arterial endothelial cell markers of the artery, but venous alteration is not well-reflected. HCC is mainly supplied by hepatic arteries, while in the normal liver parenchyma, regenerative and dysplastic nodules are mainly supplied by the portal vein. The main drainage vessels of hepatocellular nodules change from hepatic veins to hepatic sinusoids, and then to portal veins during hepatocarcinogenesis (7). To date, few studies have focused on comprehensive functional vessel network properties. Therefore, the understanding of vascular connectivity needs to be further elucidated.

The increasing availability of high-resolution micro-computed tomography (Micro-CT) allows to precisely identify and describe the pathological processes of hepatic vessels (8–10). Micro-CT enabled cirrhogenic features to be extracted at multiple scales, portraying the impact of cirrhosis on the hepatic vasculature (11).

Graph analysis approach is a valuable tool for analyzing topological properties of complex network connections, and it has been widely used in neuroimaging to explore brain function (12–14). In terms of liver imaging, a recent study successfully assessed vascular connectivity in cirrhosis using graph analysis of vascular images obtained with hepatic dynamic contrast–enhanced (DCE) ultrasonography (US). The results demonstrated that graph modeling of vascular connectivity and subsequent graph analysis may enable reflection of the degree of organization of hepatic microvascular network correlated to the severity of portal hypertension (15). In addition, the combination of graph analysis and Micro-CT could exhibit the vascular alterations during cirrhogenesis in the rat (11), and it has also been used in the vascular analysis of glioblastoma xenografts (16). Therefore, we hypothesize that graph analysis may also be feasible for assessment of HCC vascular connectivity.

In the present study, we aimed to investigate vascular topological connectomes of HCC in orthotopic xenograft model using graph analysis based on Micro-CT image.

## Materials and Methods

### Orthotopic HCC Xenograft Model

This study was reviewed and approved by the ethics committee of the Jinsan Hospital of Fudan University. Twenty Balb/c male nude mice (Weitong Lihua Experimental Animal Technology Company, Beijing, China) at 4 to 6 weeks of age and weighing 18 to 20 g each were included in this study. They were randomly divided into the HCC group (n=10) and the normal control group (n=10). For the HCC group, MHCC97H cells (2×10^6^/0.2 ml/site) were inoculated subcutaneously into the left axilla. When the tumor grew up to 1 cm in diameter, it was removed and cut into tumor blocks with a volume of 1 mm^3^, which implanted into the left lobe of the liver (17). The animals were continued to feed for 21 days.

### Vascular Casting

The animals were anesthetized with an intraperitoneal injection of 1% sodium pentobarbital (0.01 ml/g) followed by 3 ml sodium heparin solution (1250 u/ml). After ligament of bilateral superior vena cava and inferior vena cava near the heart, a drainage channel was established using a 26G indwelling needle from the left ventricle to the aorta, and inferior vena cava. Then, 20 ml of heparinized saline (50 u/ml) was injected followed by perfusing 5 ml of 10% formalin to fixed blood vessels using a laboratory syringe pump at a rate of 2 ml/min. Therewith, 5.6 ml of Microfil (Flow-Tech) was perfused at a rate of 0.5 ml/min. The livers were excised and fixed in 10% formalin for 24 hours to prepare for Micro-CT scanning (18, 19).

### Micro-CT Scanning

Micro-CT scanning was performed using a high-resolution cone-beam Micro-CT scanner (Quamtum GX). The scan parameters are as follows: source voltage of 90 KV, source current of 88 mA, voxel size of 4.5 × 4.5 × 4.5 μm^3^, field of view of 36×25 mm^2^, reconstruction matrix of 512×512, scanned 360 degrees. The average scan duration was 14 min. CT images were performed with flat-field and dark-field correction, and the image smoothing method was utilized to suppress noise.

### Vascular Segmentation

**Figure 1:**
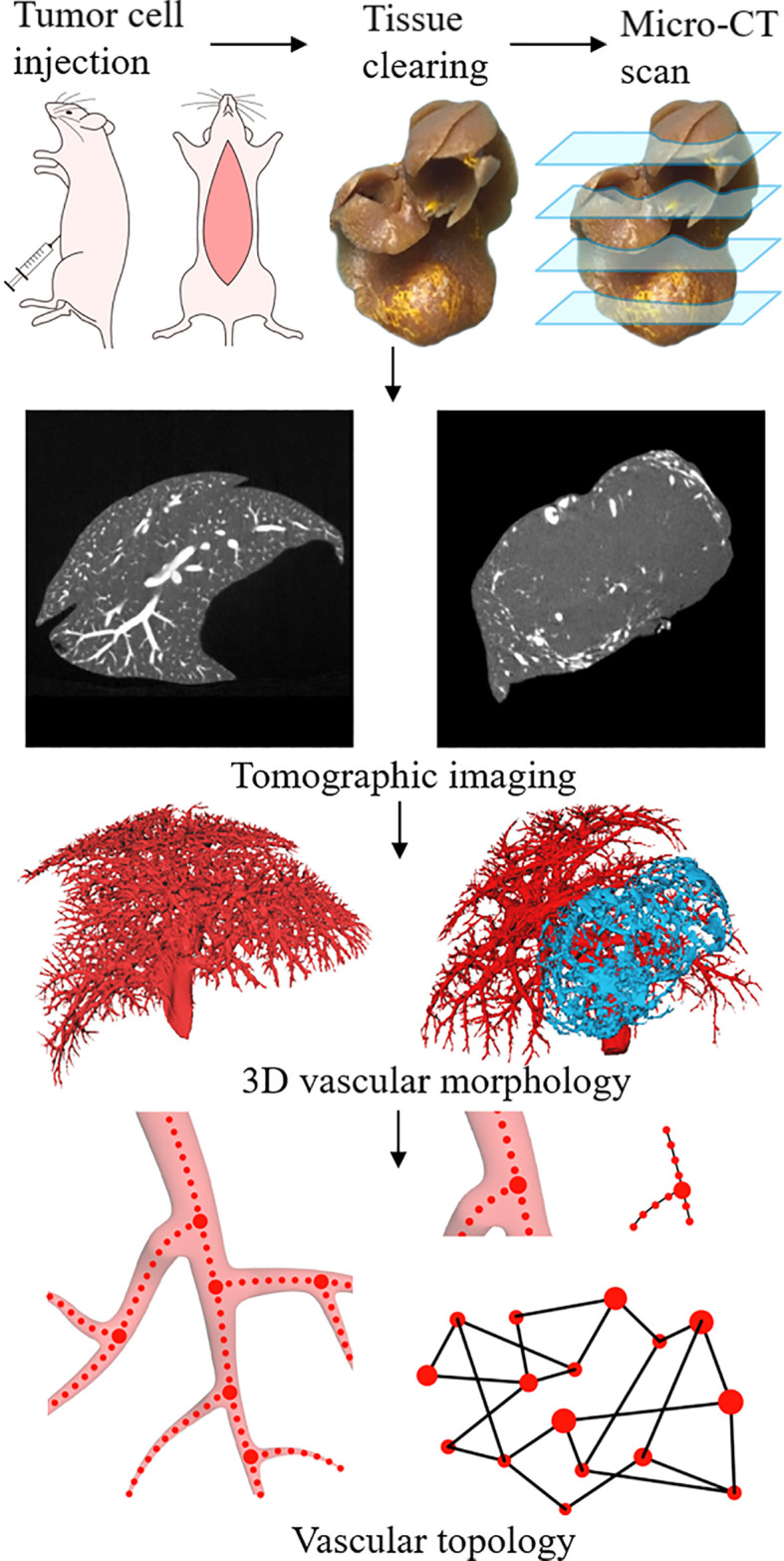
Schematic illustration of experimental procedures, including tumor cell injections, tissue perfusion and clearing, and Micro-CT scan. Extraction of vascular network based on the 3D structure reconstructed from tomographic images. Using the vascular skeleton, the network topology is studied.

**Figure 2:**
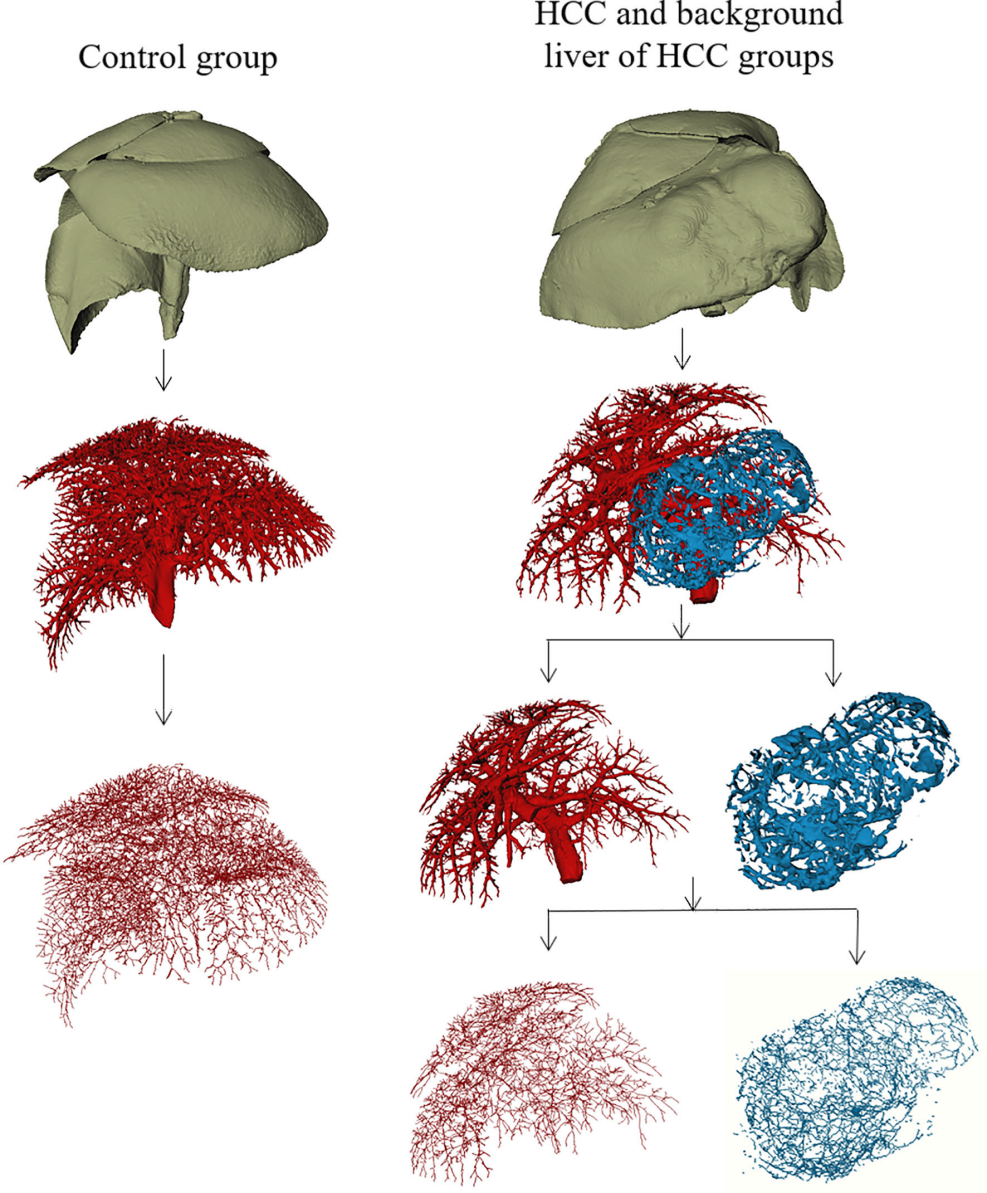
Macroscopic 3D reconstructions and centerlines of the control group, the tumor region of HCC group, and background liver of HCC group.

The algorithm from the input micro-CT data to the output of the final liver vascular segmentation results was roughly divided into three steps: 1) data preprocessing, including extracting liver region, gray map transformation, and vascular enhancement filtering (20); 2) initial vessel segmentation using the threshold method and post-processed with region growing algorithm; and 3) vessel centerlines extraction using previous vascular skeletonization algorithm (21), to generate a specialized vascular network while preserving topological and geometrical conditions (**Figures 1** and **2**).

### Graphy Analysis

The centerline voxels of vessels were used to generate the specialized network according to the number of centerline voxels in their 26 neighbors. Every single bifurcation point (more than two neighbors) or terminal point (only one neighbor) represented a node in the network; the edge connected two nodes when they were connected by voxels with only two neighbors. Noting that multiple voxels might have more than two neighbors at a bifurcation, we performed the connected components analysis on these voxels to ensure that one bifurcation corresponded to one node. The constructed network can be further encoded into an adjacency matrix and the characteristics of clustering coefficient (CC), network structure entropy (NSE), and average path length (APL) can be measured (20).

We computed the clustering coefficient (CC) as a measure of network cohesiveness and organization, in which highly organized networks possess higher CC values, whereas random networks have CC values near to zero. The definition of CC is as follows:

(1) $$CC=\frac{1}{N}\sum_{i=1}^{N}\frac{2e_{i}}{k_{i}(k_{i}-1)}$$

here, *e_i_* is the number of edges linking the neighbors and node *i*, *k_i_* is the number of neighbors, and *N* is the number of nodes within the network. By the definition, 0 ≤ *CC* ≤ 1.

The average path length of the network is the mean value of the shortest distance between each pair of nodes. We calculated the average path length (APL) as a measure of network efficiency, as longer APL means the lower working efficiency of the organization network (22). Considering all the couples, it can be calculated as:

(2) $$APL=\frac{N(N-1)}{2}\sum_{1\leq i<j\leq N}d_{ij}$$

here, *d_ij_* is the minimum number of edges linking nodes *i*, *j* , and *N* is the number of nodes within the network.

Entropy is a physical aspect of complex system structure, and quantifying network structure entropy (NSE) enables us to better understand the structural complexity and randomness of a network, which can be defined as:

(3) $$E=-\sum_{i=1}^{N}I_{i}\cdot \ln I_{i}$$

(4) $$I_{i}=\frac{D_{i}}{\Sigma_{i=1}^{N}D_{i}}$$

here, *I_i_* is the importance of node *i* and *D_i_* is the degree of the node *i*, $E_{max}=\frac{1}{N}$ and *E_min_* = 0 (16, 17). To eliminate the impact of nodes numbers within the network, the normalized network structure entropy *NSE* ∈ [0,1] is carried out:

$$NSE=\frac{E-E_{min}}{E_{max}-E_{min}}$$

### Statistical Analysis

Statistical analyses were performed with the commercially available SPSS Version 16.0J package (SPSS Inc, Chicago, IL, USA) and Graphpad Prism 6.0 (GraphPad Software Inc, La Jolla, CA, USA). Kolmogorov-Smirnov analysis was used to test for normality. Continuous variables (CC, NSE and APL of the control group and the tumor region of HCC group; CC and APL of the background liver of HCC group) showing normal distribution were expressed as the mean value with standard deviation, and data showing non-normal distribution (NSE of the background liver of HCC group) were expressed as the median with 25th and 75th percentiles of the interquartile range (IQR). Statistical comparisons were performed using the Student’s *t*-test or the Mann-Whitney U test. *P* values less than 0.05 were considered to indicate a significant difference.

## Results

One animal failed to perfuse and was excluded in the control group; a total of 19 animals were finally included in the study. From the 3D reconstructed images, it could be observed that the normal liver vessels were evenly distributed, with a natural course, and without distortion. The tumor region of HCC group showed irregular vascular morphology, with some abnormal expansion and distortion, and unevenly distributed, with sparse vessels inside the tumor, which may be related to the necrosis inside the tumor. In addition, it could be directly visualized that the vessels in the background liver of HCC group were significantly sparser than those in the control group (**Figure 2**).

### Scale of the Vascular Network

**Figure 3:**
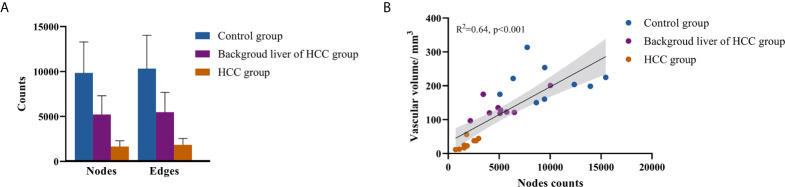
Scale of vascular network. **(A)** Nodes and edges counts; **(B)** relationship between nodes counts and segmented vascular volume.

The mean node count of vascular network in control group, background liver, and HCC group were 9839, 5220, and 1654, and the mean edge count were 10336, 5478, and 1846 (**Figure 3A**). No significant difference between the node and edge count was observed. Despite existing differences in the scale of individual networks, a linear relationship between node count and segmented vascular volume was detected (**Figure 3B**). We therefore consider differences in network scale to be individual differences, which have been averaged over the network when calculating the network features.

### Vascular Connectivity of Tumor Region of HCC and Control Group

**Figure 4:**
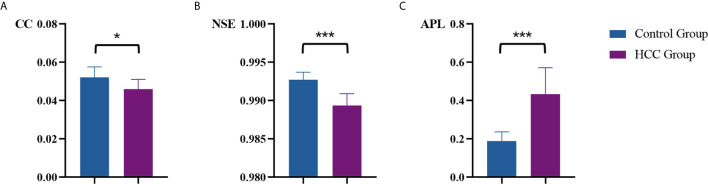
Comparison of vascular parameters **(A)** CC, **(B)** NSE and **(C)** APL of the control group and the tumor region of HCC group. *p < .05, ***p < .001.

**Table 1** summarized vascular network connectivity properties of the normal control group and the tumor region of HCC group. Compared with the normal control group, the tumor region of HCC group showed lower CC and NSE and increased APL (*P*<0.05). These indicated disrupted organization, decreased complexity, and lower efficiency of the HCC vascular network (**Figures 4A–C**).

**Table 1:** Comparison of vascular network connectivity of tumor region of HCC group and normal control group.

	CC	NSE	APL
Control group	0.052 ± 0.006	0.9927 ± 0.0010	0.188 ± 0.049
Tumor region of HCC group	0.046 ± 0.005	0.9894 ± 0.0015	0.433 ± 0.138
*P* value	0.026	<0.001	<0.001

### Vascular Connectivity of Background Liver of HCC Group and Control Group

**Figure 5:**
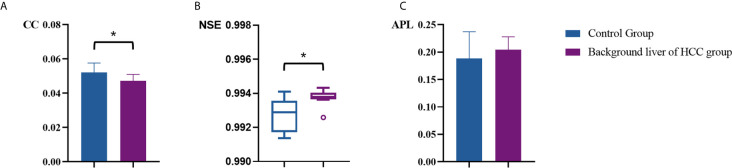
Comparison of vascular parameters **(A)** CC, **(B)** NSE and **(C)** APL of the control group and the background liver of HCC group. *p < .05.

**Table 2** summarized vascular network connectivity properties of the normal control group and the background liver of HCC group. Compared with the normal control group, the background liver of HCC group showed lower CC and slightly increased NSE (*P*<0.05). These indicated disrupted organization and increased complexity. No significant difference was identified for APL between the two groups, indicating maintained normal work efficiency (**Figures 5A–C**).

**Table 2:** Comparison of vascular connectivity of background liver of HCC group and normal control group.

	CC	NSE	APL
Control group	0.052 ± 0.006	0.9927 ± 0.0010	0.188 ± 0.049
Background liver of HCC group	0.047 ± 0.004	0.9938 (0.9936~0.9940)	0.205 ± 0.023
*P* value	0.041	0.035	0.385

## Discussion

Our study demonstrates the feasibility to investigate vascular topological connectivity of HCC using graph analysis based on micro-CT, we tends to show abnormal vascular connectivity compared to normal liver. This may provide new insights into the HCC angiogenesis study.

Intratumoral microvessel heterogeneity of HCC has been well validated. The main hallmarks of tumor angiogenesis are considered to be an elevated vessel density, dilated vessel radii, higher tortuosity, and decreased branching lengths (23), lacking a description of the topological properties of vascular network. Afferent and efferent vessels of HCC come to differ during hepatocarcinogenesis, and whole vascular network characteristics significantly determine efficient blood perfusion, oxygen, and nutrients transportation (24). Normal liver vasculature typically follows a hierarchical arterio-venous branching scheme, with blood flowing through thick arteries and portal veins, successively branching into sinusoids and draining the tissue in a similarly organized hepatic venous system. Forming efficient transport networks, normal vessel constructs are inherent to tree-structured arterial and venous parts, interwoven by dense, regular capillary beds (25–27).

In the present study, we established the orthotopic HCC xenograft model that mimics human HCC well in morphology and biological behavior. We characterized the entire perfused vascular systems in normal mice liver and HCC xenografts using basic geometric and network theoretical measures, and graph-based quantification can also provide detailed vascular multi-scale topological parameters. Our research observed that vascular connectivity of HCC showed reduced CC and NSE and longer APL, and we considered these changes indicated a disrupted organization, decreased complexity, and lower efficiency network for HCC. Impaired vascular connectivity may lead to hypoxia microenvironment, which enhances proliferation, angiogenesis, metastasis, chemoresistance, and radioresistance. Target vascular topology may be a potential strategy for the evaluation of anti-angiogenesis treatment efficacy.

Furthermore, our study also found abnormal vascular topological connectivity in the background liver of HCC xenografts. In the micro-CT images, vessel branching patterns and dimensions of background liver differ from the normal liver. Graph analysis showed lower CC and slightly increased NSE in the background liver, indicating a tendency to additional random alterations in the background liver vascular network of HCC, which shows disrupted organization and increased complexity. This may reflect the interaction between the tumor and background liver during hepatocarcinogenesis.

Our study has considerable limitations. Firstly, when distinguishing the tumor and liver parenchyma, we manually delineate the tumor boundaries. Some vessels in tumors might not connect to the center vessel trees that resulted in inadequate filling during the perfusion process. Secondly, it should be noted that vessels in the context of corrosion casts relate to a cast of the vessel lumen space but not the actual vessel (which includes additional layers of cells and proteins). Finally, the small sample size is also one of the limitations of this study.

In conclusion, graph-based approach allows quantification of vascular connectivity of HCC. Disrupted vascular topological connectivity exists in the tumor region as well as the background liver of HCC.

## Data Availability Statement

The original contributions presented in the study are included in the article/**Supplementary Material**. Further inquiries can be directed to the corresponding authors.

## Ethics Statement

The animal study was reviewed and approved by the ethics committee of the Jinsan Hospital of Fudan University.

## Author Contributions

All authors listed have made a substantial, direct, and intellectual contribution to the work, and approved it for publication.

## Funding

This work was supported by the National Natural Science Foundation of China (DX: No. 81371520, 81501433 and 81971583) and Shanghai Municipal Science and Technology Major Project (No. 2018SHZDZX01). The funders had no role in study design, collection, analysis, and interpretation of data, and in the writing the manuscript.

## Conflict of Interest

The authors declare that the research was conducted in the absence of any commercial or financial relationships that could be construed as a potential conflict of interest.
