# Supplementary material for: Assessment of Vascular Network Connectivity of Hepatocellular Carcinoma Using Graph-Based Approach
Source: Front Oncol. 2021 Jul 6;11:668874. doi: 10.3389/fonc.2021.668874 (PMC8290165; doi:10.3389/fonc.2021.668874)
Supplement: Supplementary file 1 [file DataSheet_1.pdf]

1.Vascular Segmentation

(1) Determine the tumor region on Micro-CT images

Regions of interest were manually delineated along the edge of the tumors in each slice in order to derive the whole tumor volume from the liver. This work was finished in cooperation by one radiologist and one biomedical engineer with 3 years of experience in abdominal imaging interpretation and post-procession.

(2) Preprocessing

a. Extracting liver region: a relatively low threshold had been adopted to separate the liver from the background, which helped to reduce the computational effort.

b. Gray map transformation: the normalization was completed with the z-score method[1] which was measured in terms of standard deviations from the mean. This process helped to reduce the impact of inter-individual differences in the gray values on further processes.

c. Vessel enhancement: Vascular enhancement filtering was performed using Sato's method, which helped to enhance the vascular region. The enhanced result was superimposed on the original image for subsequent segmentation.

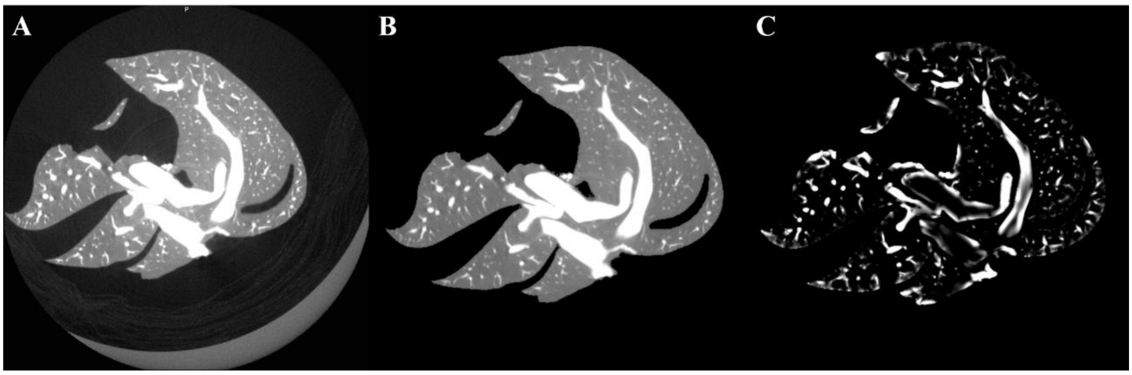

Figure 1. Preprocessing of Micro-CT images; A) the original image; B) extracted liver region; C) vascular enhancement result.

(3) Segmentation

Since Vessels and background were divided into distinct gray scale ranges due to the attenuation effect of the Microfil, the vascular voxels were automatically extracted by the global thresholding method (Ostu's method)[2], and followed with the region-growing segmentation method. The region growing method was used to ensure the 3-

dimensional integrity of the vascular three after the threshold segmentation.

(4) Centerline extraction

The centerline extraction was finished using the established method[3]. This method used the segmented vessels as input and output the centerlines.

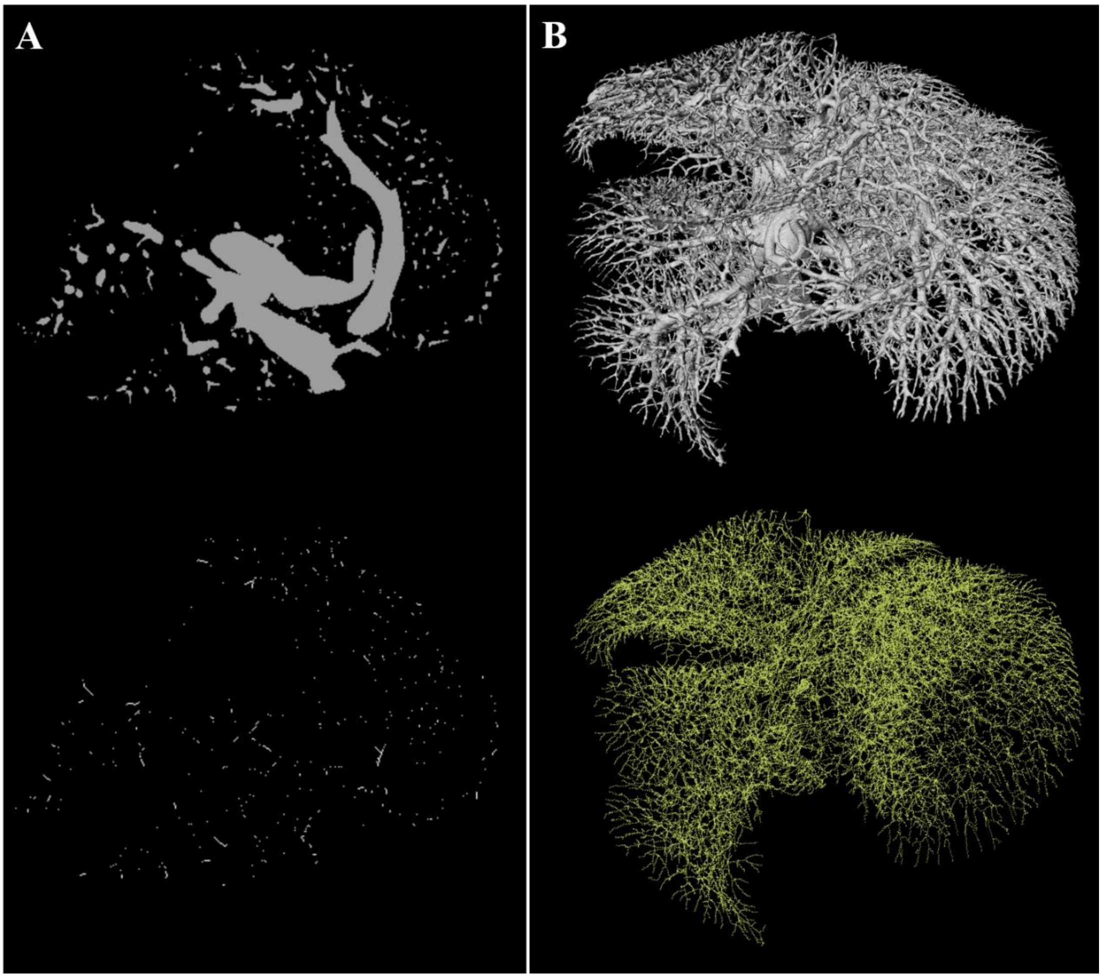

Figure 2. Segmentation (up) and centerline extraction (down) result; A) result on one slice of images; B) 3D reconstruction result.

2.Graphy analysis

The centerline of vessels was used to generate the specialized vascular network: every single bifurcation point or terminal point within the centerline represented a node in the network; the edge of the network linked two nodes if they were connected directly and no other nodes between them. Once the network was constructed, we encoded the network into an adjacency matrix and evaluate the corresponding characteristics: clustering coefficient, network structure entropy, and average path length as described

in the main text.

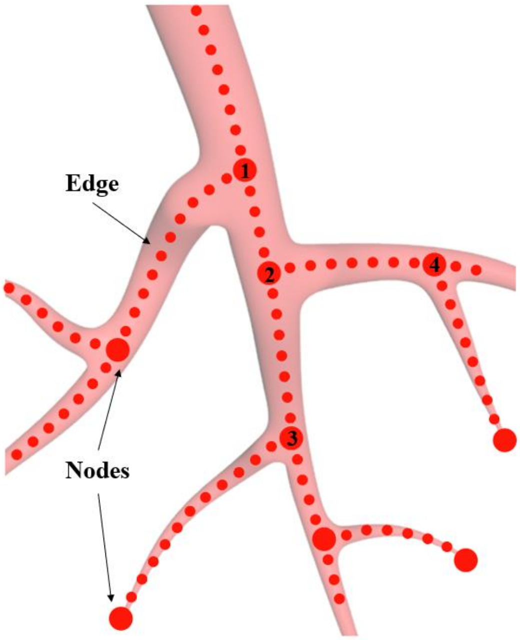

The adjacency matrix

	Node1	Node2	Node3	Node4
Node1	0	1	0	0
Node2	1	0	1	1
Node3	0	1	0	0
Node4	0	1	0	0

Figure 3. Schematic representation of how the vascular network was generated. Nodes of the vascular network were the bifurcation points or terminal points among the centerline points. Edges were determined by whether the two nodes were directly connected though other centerline points (no other nodes between them). The adjacency matrix digitally represented the adjacency of the vascular network, where 1 indicated that two nodes were adjacent, and 0 for not adjacent.

Reference

[1] R. Zheng, L. Wang, C. Wang, X. Yu, W. Chen, Y. Li, W. Li, F. Yan, H. Wang, and R. Li, Feasibility of automatic detection of small hepatocellular carcinoma (≤2 cm) in cirrhotic liver based on pattern matching and deep learning. Physics in medicine and biology 66 (2021).

[2] N. Otsu, THRESHOLD SELECTION METHOD FROM GRAY-LEVEL HISTOGRAMS. Ieee Transactions on Systems Man and Cybernetics 9 (1979) 62-66.

[3] P. Kollmannsberger, M. Kerschnitzki, F. Repp, W. Wagermaier, R. Weinkamer, and P. Fratzl, The small world of osteocytes: connectomics of the lacuno-canalicular network in bone. New Journal of Physics 19 (2017).
