# Supplementary figures and images for: Assessment of Vascular Network Connectivity of Hepatocellular Carcinoma Using Graph-Based Approach
Source: Front Oncol. 2021 Jul 6;11:668874. doi: 10.3389/fonc.2021.668874 (PMC8290165; doi:10.3389/fonc.2021.668874)

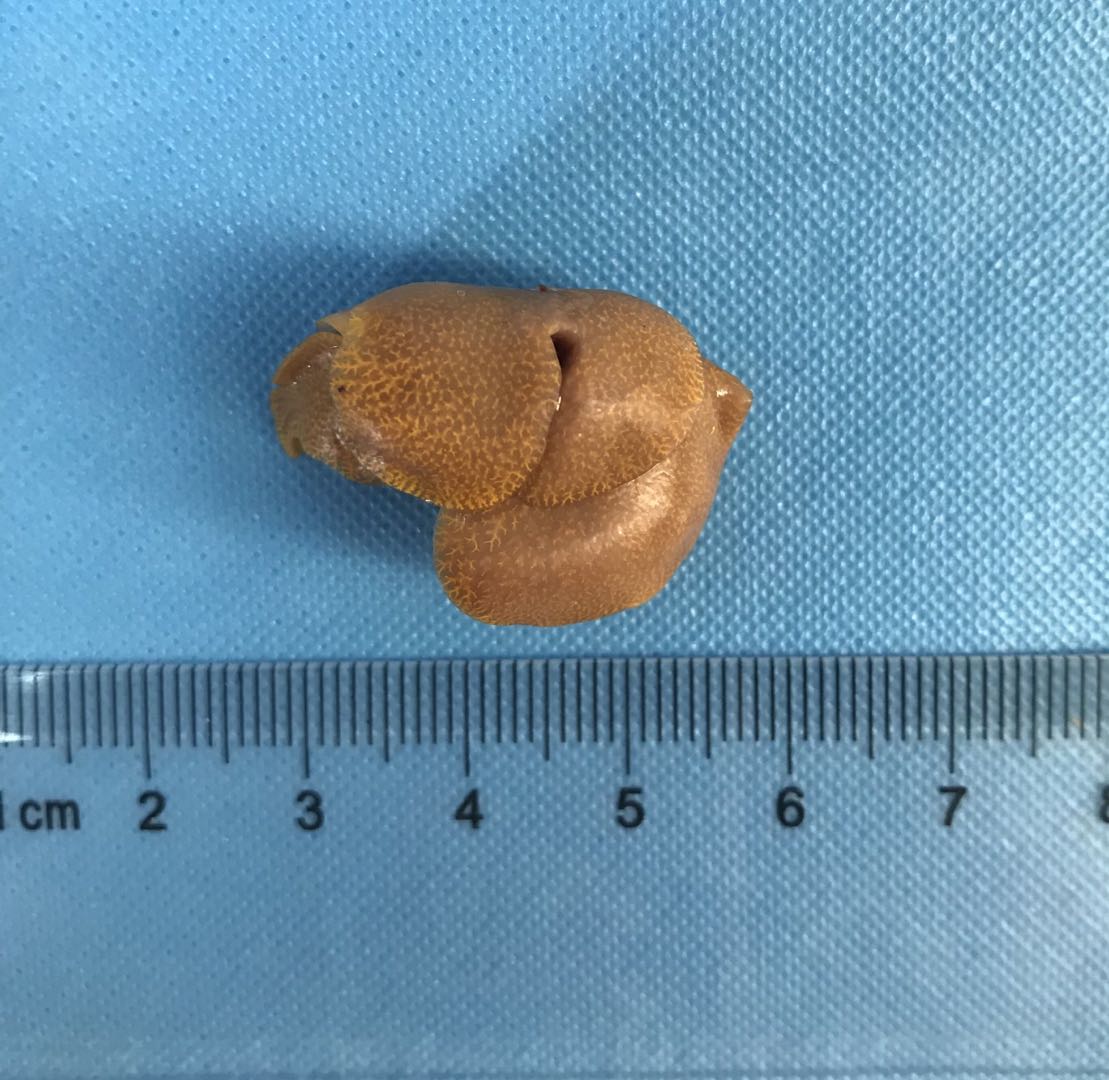

Supplement: Supplementary file 2 [file Image_1.jpeg]

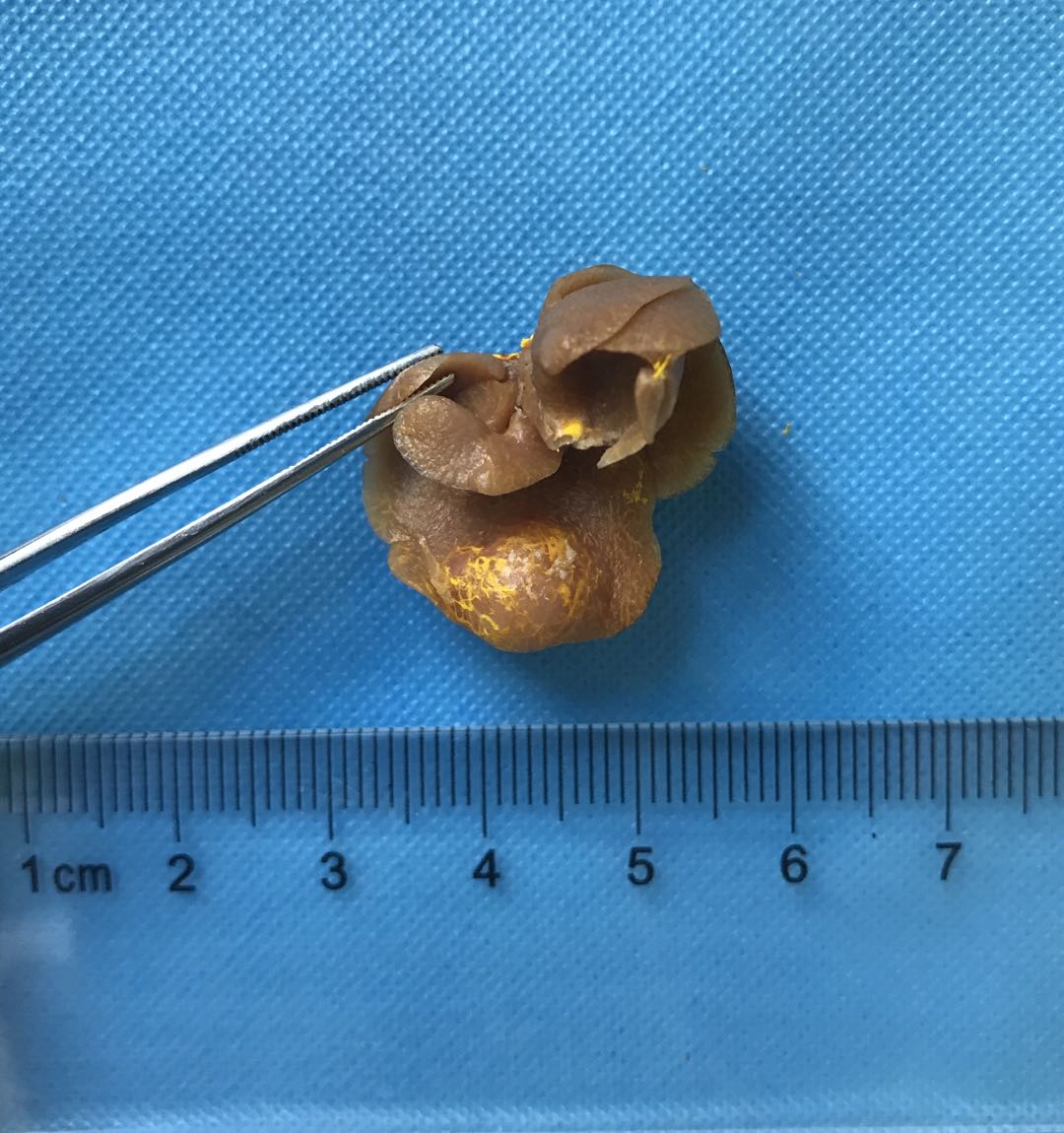

Supplement: Supplementary file 3 [file Image_2.jpeg]
